# Exosomal Proteome Profiling: A Potential Multi-Marker Cellular Phenotyping Tool to Characterize Hypoxia-Induced Radiation Resistance in Breast Cancer

**DOI:** 10.3390/proteomes1020087

**Published:** 2013-08-09

**Authors:** Stefani N. Thomas, Zhongping Liao, David Clark, Yangyi Chen, Ramin Samadani, Li Mao, David K. Ann, Janet E. Baulch, Paul Shapiro, Austin J. Yang

**Affiliations:** 1 Department of Pharmacology and Molecular Sciences, Johns Hopkins University School of Medicine, Baltimore, MD 21205, USA; E-Mail: sthoma92@jhmi.edu; 2 Eli Lilly and Company, Indianapolis, IN 46285, USA; E-Mail: zliao001@umaryland.edu; 3 Greenebaum Cancer Center, University of Maryland School of Medicine, Baltimore, MD 21201, USA; E-Mails: djclark81@gmail.com (D.C.); yangyic@gmail.com (Y.C.); pshapiro@rx.umaryland.edu (P.S.); 4 Division of Oncology, University of Maryland School of Dentistry, Baltimore, MD 21201, USA; 5 Department of Pharmaceutical Sciences, University of Maryland School of Pharmacy, Baltimore, MD 21201, USA; E-Mails: rsama002@umaryland.edu; 6 Oncology and Diagnostic Sciences, University of Maryland School of Dentistry, Baltimore, MD 21201, USA; E-Mail: LMao@umaryland.edu; 7 Department of Molecular Pharmacology, Beckman Research Institute, City of Hope, Duarte, CA 91010, USA; E-Mail: dann@coh.org; 8 Irell and Manella Graduate School of Biological Sciences, Beckman Research Institute, City of Hope, Duarte, CA 91010, USA; 9 Department of Radiation Oncology, University of California, Irvine, CA 92697, USA; E-Mail: jbaulch@uci.edu; 10 Department of Anatomy and Neurobiology, University of Maryland School of Medicine, Baltimore, MD 21201, USA

**Keywords:** hypoxia, radiation, breast cancer, tumor microenvironment, exosomes, proteomics

## Abstract

Radiation and drug resistance are significant challenges in the treatment of locally advanced, recurrent and metastatic breast cancer that contribute to mortality. Clinically, radiotherapy requires oxygen to generate cytotoxic free radicals that cause DNA damage and allow that damage to become fixed in the genome rather than repaired. However, approximately 40% of all breast cancers have hypoxic tumor microenvironments that render cancer cells significantly more resistant to irradiation. Hypoxic stimuli trigger changes in the cell death/survival pathway that lead to increased cellular radiation resistance. As a result, the development of noninvasive strategies to assess tumor hypoxia in breast cancer has recently received considerable attention. Exosomes are secreted nanovesicles that have roles in paracrine signaling during breast tumor progression, including tumor-stromal interactions, activation of proliferative pathways and immunosuppression. The recent development of protocols to isolate and purify exosomes, as well as advances in mass spectrometry-based proteomics have facilitated the comprehensive analysis of exosome content and function. Using these tools, studies have demonstrated that the proteome profiles of tumor-derived exosomes are indicative of the oxygenation status of patient tumors. They have also demonstrated that exosome signaling pathways are potentially targetable drivers of hypoxia-dependent intercellular signaling during tumorigenesis. This article provides an overview of how proteomic tools can be effectively used to characterize exosomes and elucidate fundamental signaling pathways and survival mechanisms underlying hypoxia-mediated radiation resistance in breast cancer.

## 1. Introduction

In the United States, breast cancer is the most common non-skin cancer and the second leading cause of cancer-related death in women. It is estimated that women have a 12% risk of developing breast cancer in their lifetime. Although targeted therapies such as the use of Trastuzumab have been shown to improve survival in the adjuvant treatment of HER-positive breast cancer [1], resistance to radiation therapy and chemotherapy in locally advanced and metastatic disease are significant challenges and causes of mortality.

It has been shown that 40% of all breast cancers and 50% of locally advanced breast cancers contain hypoxic regions where the effectiveness of radiation and chemotherapy is reduced [2]. Hypoxic tumors have more aggressive phenotypes and are associated with poor patient outcomes in several types of cancer [3]. Thus, there is a need to develop therapeutic strategies to overcome the challenges that are related to tumor hypoxia.

There has been recent interest in the role of exosomes in mediating the cross-talk between various cell types [4,5,6,7,8,9]. These cell-secreted microvesicles are highly enriched in biological fluids such as blood plasma and contain effectors of carcinogenesis such as growth factors, cytokines, mRNA, microRNA and bioactive lipids. A positive correlation between the abundance of secreted exosomes and cancer stage and progression has been demonstrated [10], and several comprehensive reviews of the role of exosomes in cancer development, metastasis and drug resistance have been published recently [11,12,13,14]. The factors and stimuli that regulate exosome secretion are not completely understood, but roles have been reported for ionizing radiation [15,16] in addition to p53 [17], ceramide synthesis [18], calcium signaling [19] and acidosis [20].

**Figure 1:**
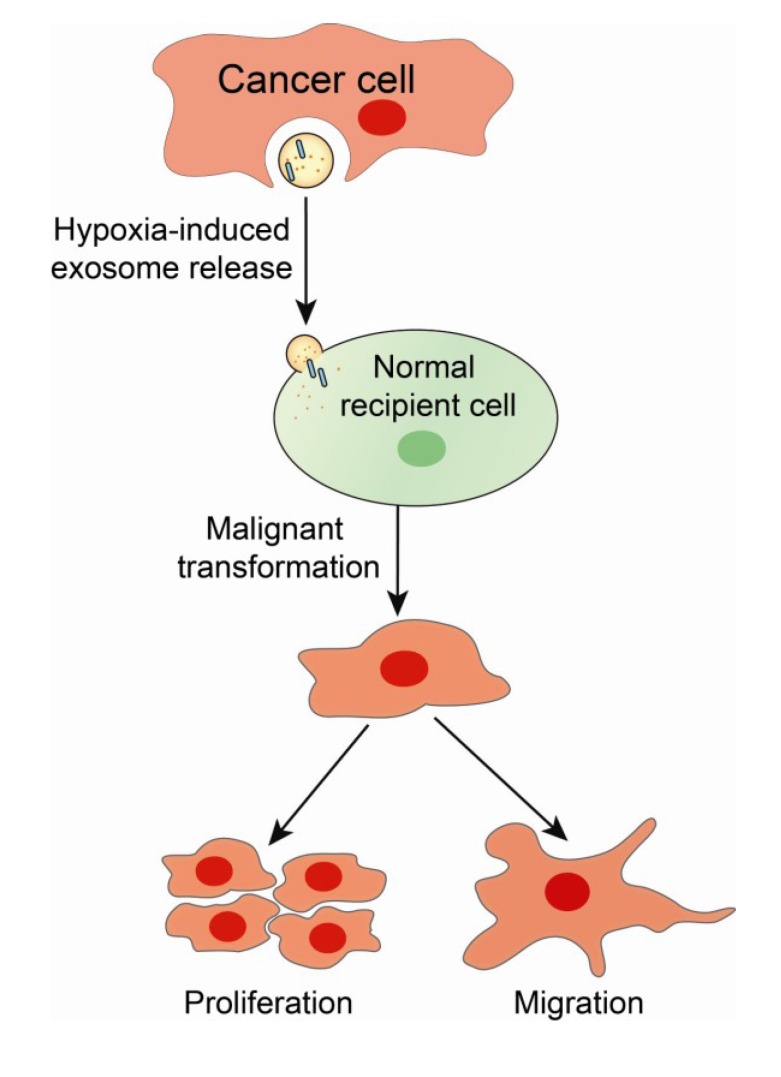
The hypoxia-induced release of exosomes from cancer cells is hypothesized to result in the malignant transformation and subsequent proliferation and migration of normal recipient cells.

The hypoxia-induced release of exosomes from cancer cells has been hypothesized to cause the malignant transformation of normal recipient cells, which results in malignant cell proliferation and migration [4,10,21] as shown schematically in Figure 1. It is well known that hypoxic tumor cell-derived exosomes promote angiogenic signaling [8,21], and increased exosome release has been demonstrated under hypoxic conditions [22,23]. Although many of these studies were conducted *in vitro* using a murine model, the results suggest several important functional implications of the role of exosomes in the hypoxic tumor microenvironment. *In vitro* data suggest an association among hypoxia, exosome-mediated signaling and invasive tumor phenotypes [8,11,24], and there has been increased interest in determining whether hypoxia is able to stimulate tumor progression through altered exosome release. For example, King *et al*. have shown that breast cancer cells grown under hypoxic conditions release greater numbers of exosomes than cells grown under normoxia [24]. Furthermore, Kucharzewska *et al*. have demonstrated that the proteome and mRNA profiles of exosomes closely reflect the oxygenation status of donor glioma cells and patient tumors, and that exosomal signaling is a potentially targetable inducer of hypoxia-dependent intercellular signaling during tumor development [4]. For this reason, proteomic profiling of tumor exosomes circulating in cancer patient plasma or serum has the potential to provide functional diagnostic markers of disease without the invasiveness of biopsy procedures [25,26]. An important consideration with this approach is that tumor exosomes are only a small subpopulation of the microvesicles that are present in the blood, which therefore necessitates the development of selective sorting procedures such as those that include the use of affinity agents.

A better understanding of the role for exosome signaling in cancer cell communication may allow for the development of novel strategies to overcome the therapeutic challenges related to tumor hypoxia. New deep coverage proteomic approaches that involve mass spectrometry instrumentation with high sensitivity, resolution and mass accuracy, and more stringent exosome isolation and purification strategies will yield valuable insight into the identification and function of exosomes that are uniquely released under hypoxic conditions and contribute to radiation resistance in breast cancer. In this paper we will describe the role of hypoxia in breast carcinogenesis, basic exosome biology, and the potential for manipulating exosome-mediated intercellular signaling to increase the efficacy of breast cancer radiotherapy.

## 2. Hypoxia and the Tumor Microenvironment—Mechanisms of Radiation Resistance

**Figure 2:**
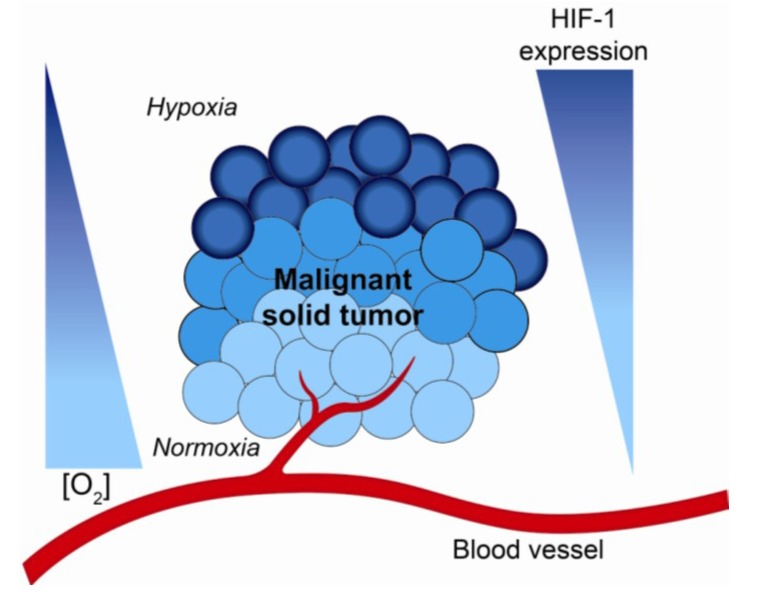
Spatial relationship between a blood vessel, hypoxic conditions and a malignant solid tumor in the context of O_2_ and HIF-1 concentrations. Normoxic conditions, which are defined by oxygen concentrations above 5%, exist in regions that are less than 70 µM from tumor blood vessels. In these regions, HIF-1 is rapidly degraded, or it has a low level of expression. Hypoxic conditions exist at distances greater than 100 µM from tumor blood vessels. In these regions, HIF-1 expression is increased and O_2_ concentrations are decreased, which creates a hypoxic tumor microenvironment.

Approximately 40% of all breast cancers contain hypoxic microenvironments where chemotherapy and radiation are less effective [2]. In normal tissues, the oxygen supply is in equilibrium with the metabolic requirements; however, tumor cells often have an increased demand for oxygen. Tumor cells adapt to exist in these hypoxic microenvironments by up-regulating pro-survival mechanisms, the majority of which are coordinated by the transcription factor hypoxia inducible factor-1 α (HIF-1α; Figure 2) [27].

Hypoxia is a main force that drives cancer cells to adopt a more radiation resistant and invasive phenotype [28]. Chronic hypoxia contributes to radiation resistance by preventing the activation of the G1/S cell cycle checkpoint, thereby allowing DNA replication errors to accumulate and fostering genomic instability [29]. By hindering the normal progression of the cell cycle, hypoxia is directly associated with rendering cells resistant to drugs that target cell proliferation.

The ability of molecular oxygen to influence the biological effect of ionizing radiation is known as the oxygen effect [30]. Hypoxic stimuli trigger changes in DNA repair and cell death/survival signaling pathways [31]. Although the precise mechanism of action of the oxygen effect is unknown, it is widely accepted that oxygen acts at the level of free radical generation [32]. Ionizing radiation exposure induces ionization events within a few nanometers of genomic DNA in target cells and produces free radicals that cause DNA damage [33]. Oxygen oxidizes the DNA radicals and can cause the damage to become fixed in the genome. Conversely, in the absence of oxygen, as is the case in hypoxic environments, the production of DNA radicals is reduced, thereby decreasing their overall effect. DNA damage, including irreparable double strand breaks, is significantly less severe in the absence of molecular oxygen [34,35], resulting in the hypoxia-related radiation resistance of cancer cells. However, it has recently been shown that hypoxia up-regulates the nonhomologous end joining pathway that is involved in the DNA repair of irradiated cells, which could contribute to the radiation resistance of hypoxic A431 epithelial carcinoma cells [36]. Nonhomologous end joining and homologous recombination are the major DNA double strand break repair mechanisms; however, DNA damage response consists of several pathways, some of which act independently and others that do not [37]. Hence, hypoxia can affect DNA repair via multiple mechanisms.

In breast cancer cells, hypoxia has been shown to promote the release of exosomes, which is partly mediated by HIF-1α [24]. In their study, King *et al*. demonstrated that the exposure of three different breast cancer cell lines to moderate (1% O_2_) and severe (0.1% O_2_) hypoxia resulted in significant increases in the number of exosomes in the conditioned media from these cells, as determined by Western blot detection of the exosomal protein CD63 [24]. Furthermore, they showed that the activation of hypoxic signaling by a HIF-1α hydroxylase inhibitor resulted in a significant increase in exosome release. They also demonstrated that the transfection of cells with HIF-1α siRNA before hypoxic exposure prevented the hypoxia-induced enhancement of exosome release. Taken together, these results suggest a functional link between HIF-1α and the hypoxic response in breast cancer cells.

Interestingly, we have identified a novel non-HIF-1α-mediated response to hypoxia in breast cancer cells and high-grade or recurrent breast tumors [38]. Our results demonstrated that exposure to hypoxia causes the loss of function of vacuolar protein sorting-associated protein VPS4B, which is involved in maintaining the fidelity of multivesicular body (MVB) maturation, resulting in increased breast cancer cell anchorage-independent growth and resistance to anti-EGFR, anti-MEK and genotoxicity induction treatment.

Several mechanisms of radiation resistance in tumor cells under hypoxic conditions involve the function of biological molecules including oxygen, hydrogen, miR-210, HIF-1 and N-myc downstream-regulated gene 2 (NDRG2) [39,40,41,42,43,44]. Exosomes have been implicated in the radiation resistance of tumor cells under hypoxic conditions based on their ability to shuttle RNA and protein to recipient cells [45]. Additionally, exosomes are known to have important roles in the hypoxia-mediated phenotypic alteration of the tumor vasculature [4], and more than half of the secreted proteome from hypoxic carcinoma cells can be associated with exosomes [8]. Taken together with our preliminary data that indicate a role for VPS4B in exosome production, we hypothesize that there is a mechanistic link between hypoxia, MVB function, exosome production and radiation resistance in breast cancer.

## 3. Morphological Characteristics of Exosomes

It is well established that hypoxia is directly linked to the poor prognosis of breast tumors. In addition, it has been shown that hypoxia leads to the increased production of exosomes [22,23]. Therefore, significant efforts have been made by several groups to investigate whether tumor-derived exosomes in biological fluids could potentially serve as a multiple biomarker phenotyping tool for hypoxia-induced tumorigenesis.

Exosomes are microvesicles that are known to alter the cellular phenotype of target cells and play an essential role in the growth and/or metastasis of breast cancer [46,47]. They are 40–100 nm vesicles of endocytic origin that are constitutively released by cells into the extracellular environment as a result of multivesicular endosomes fusing with the plasma membrane. Originally thought of as “garbage” vesicles that aid the removal of excess plasma membrane receptors or other cellular components, exosomes have now been shown to have a role in intercellular communication, and they are highly enriched in biological fluids such as plasma.

In addition to having a characteristic morphology, exosomes have unique protein and lipid compositions [46]. Because of their endosomal origin, all exosomes contain membrane transport and fusion proteins (GTPases, flotillin, annexins), tetraspannins (CD9, CD63, CD81, CD82), heat shock proteins (Hsc70, Hsp90), proteins that are involved in multivesicular body biogenesis (Alix, Tsg101), and lipid-related proteins and phospholipases [9,48]. Among the lipid raft-associated molecules that are enriched in exosomes are cholesterol, ceramide, sphingolipids, and phosphoglycerides [9,18,49]. The lipoprotein content of exosomal membranes renders the exosomes more stable than soluble proteins in the extracellular environment [47]. Exosomes also package and functionally deliver genetic components such as mature miRNA, mRNA, and retroviral RNA to other cells [50,51,52,53]. Macrophages have been shown to mediate the invasiveness of breast cancer through the exosome-mediated delivery of miRNA into cells to promote metastasis [7].

## 4. Isolation of Exosomes

**Figure 3:**
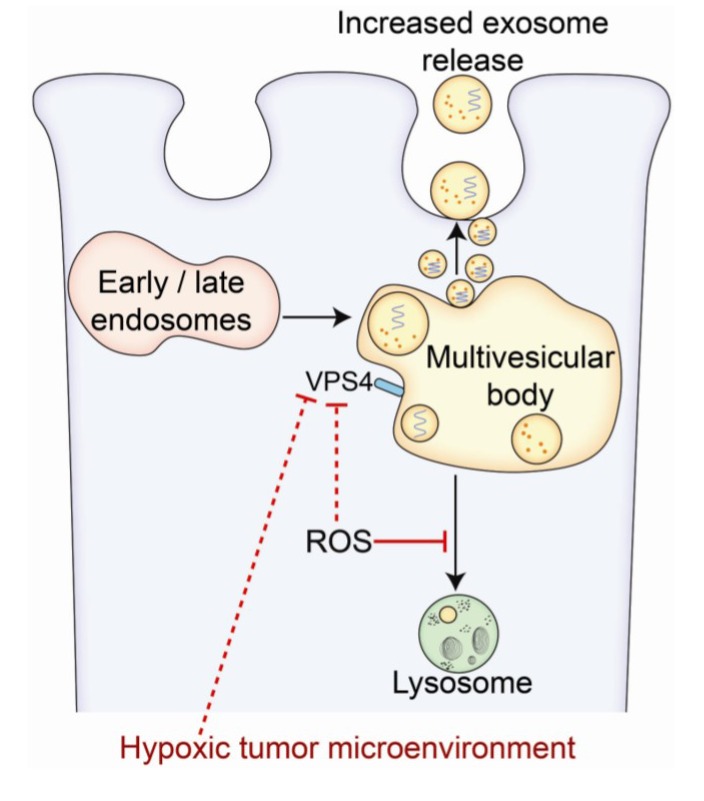
Increased exosome release resulting from hypoxia or reactive oxygen species (ROS)-induced VPS4 dysregulation.

Exosome biogenesis begins with the internalization of plasma membrane receptors that are targeted to the endosome. This inward budding of the endosomal membrane results in the formation of intraluminal vesicles (ILVs), which collect in the mature or late endosome, now termed multivesicular bodies (MVBs). MVBs can then bud and fuse with the cell membrane to be released as exosomes into the extracellular environment. The multi-protein complexes that are involved in this process include the various endosomal sorting complex required for transport (ESCRT) proteins that are involved with internalized protein recognition and the invagination of the endosomal membrane. The energy that is required for the concomitant dissociation of the ESCRT machinery and scission of the ILV from the endosomal membrane is provided by the multimeric protein complex known as the VPS4 complex. Studies from our laboratory using the HER2 over-expressing breast cancer cell line SKBR3 have demonstrated that hypoxia or reactive oxygen species (ROS)-induced VPS4 dysregulation leads to the accumulation of the ESCRT machinery in exosomes, as well as an increase in proteins that are associated with exosome biogenesis and trans-Golgi network receptor recycling [38,54,55] (Figure 3). Furthermore, under these conditions, an accumulation of exosome protein cargo was observed, which resulted in the increased relative abundance of EGFR and HER2 receptors. *In vitro*, exosomes that carry the HER2 receptor on their surface act as molecular “sponges”, binding and sequestering the monoclonal anti-HER2 antibody, Herceptin®, and reducing the amount of the antibody that is available to target the actual cancer cells for anti-cancer therapy [56,57]. It has not yet been determined whether this phenomenon occurs in patients.

Exosomes can be isolated from a variety of biological matrices. Ultracentrifugation and sucrose density gradient centrifugation are commonly accepted methods of isolating exosomes [58]. Although sequential differential centrifugation does not result in the isolation of pure populations of membrane-bound vesicles of specific sizes, sucrose gradient ultracentrifugation can be used to provide a highly enriched population of exosomes. Other methods such as free flow electrophoresis (FFE) have also been developed to isolate pure populations of exosomes [59,60]. FFE permits the separation of highly purified populations of exosomes in their native state, thereby enabling the analysis of specific proteins as they relate to biological function. Ultrafiltration and HPLC-based processes that remove cells and cellular debris using micro-filters and positive pressure have been used to generate exosome enriched samples [61]. Affinity purification methods have also been applied for the specific isolation of exosomes, and some of these methods have been developed into commercially available products. These products include anti-CD63, CD81 and CD9 antibodies that are immobilized on 96-well plates and an affinity capture method that targets specific saccharide residues on the exosome surface. In addition to these approaches, many other systems are currently being developed to enhance the efficiency and selectivity of exosome isolation. Regardless of which method is chosen, care should be taken when isolating exosomes from cultured tumor cells to avoid contamination from exosomes that are present in the fetal bovine serum of the cell culture media. Such contamination can be avoided by serum deprivation or the use of exosome-free serum. However, one of the major advantages of using a stable isotope labeling with amino acids in cell culture (SILAC)-based approach is that all exosomal proteins that are secreted by cultured tumor cells will be labeled by “heavy” amino acids such as ^13^C or ^15^N arginine and lysine. These heavy-labeled exosomal proteins could be easily separated from any normal and “light”-labeled lysine and arginine-containing proteins that are derived from fetal bovine serum. Therefore, one of the long term goals of our approach is to generate a series of SILAC-labeled tumor-derived internal standards to monitor the secretion of tumor exosomes in various biological fluids derived from cancer patients. SILAC-based quantitative proteomics is discussed in further detail in Section 7, “Proteomic analysis of the exosome proteome for the development of biomarkers”.

## 5. Exosomes as Biological Effectors and Carriers of Oncogenic Signatures in Cancer

Various strategies, such as immunohistochemistry and functional or molecular imaging, have been adopted to assess tumor hypoxia in various cancer types. However, a significant unmet need resides in the development of strategies to overcome the radiation resistance of tumor cells. The results from clinical studies have shown that cancer treatment strategies that are targeted toward hypoxia-mediated pathways necessitate a comprehensive analysis of the complex network of intercellular communication that influences the tumor microenvironment [62].

Under hypoxic conditions, cancer cells secrete exosomes that modulate their local and distant environment to facilitate tumor angiogenesis and metastasis [8]. Exosomes isolated from EGFR mutant or high-grade tumors contain many activated signaling molecules that are directly involved in EGF signaling pathways [21], and EGFR and ERK1/2 kinases are abnormally activated when cells are grown under hypoxic conditions [38]. Taken together, these studies suggest that exosomes constitute a potentially targetable mediator of hypoxia-driven tumor development and that the exosomal molecular signature could be a noninvasive biomarker to determine the oxygenation status and aggressiveness of malignant tumors.

Proteins associated with immune evasion, cell proliferation, cell invasion, metastasis, and angiogenic factors have all been identified in secreted exosomes, illustrating their potential role in tumor progression, as well as in mediating chemoresistance [63,64,65,66,67,68]. As previously discussed, exosomes have been shown to interfere with Herceptin® uptake by breast cancer cells, decreasing the drug’s therapeutic efficacy [56,57,63]. Exosomes also have a role in mediating chemoresistance. Increased expression levels of putative cisplatin export transporters MRP2, ATP7A, and ATP7B have been detected in human ovarian carcinoma cells [69]. Chen *et al*. determined that in melanoma cisplatin resistance is associated with the exosomal trapping and export of cisplatin, which contributes to multi-drug resistance [70]. Furthermore, the cytotoxic drug doxorubicin is eliminated by exosome secretion [71]. For these reasons, it has been suggested that exosomal removal by therapeutic filtration or the pharmacological targeting of exosome release could serve as an adjuvant therapy to increase treatment efficacy [63]. One such approach entails immobilized affinity agents that reside in the outer capillary region of hollow fiber plasma separator cartridges that are integrated into dialysis units or continuous renal replacement therapy machines. As the patient’s blood enters the device, plasma components that are <200 nm travel through the porous fibers and interact with the immobilized affinity agent to which target molecules are selectively adsorbed, whereas blood cells and non-bound serum components travel through the device. Ideally, affinity reagents that recognize common exosomal surface markers will bind to and trap only tumor-derived exosomes; however, the selective capture of such tumor-derived exosomes needs to be addressed. Tumor-derived exosomes contain some of the same surface markers as non-tumor-derived exosomes, albeit at a different level of expression. Thus, one of the critical aspects of the success of this method is the ability to selectively capture tumor-derived exosomes. The information that has been gleaned from the proteomic profiling of cancer cell line- and tumor-derived exosomes can be used to guide the design of affinity agents with high selectivity that are used in therapeutic filtration devices.

## 6. Proteomic Profiling of Exosomes

Improved exosome purification strategies coupled with improved mass spectrometry-based proteomic tools have significantly enhanced the analysis of the molecular composition of exosomes. Advances in mass spectrometry instrumentation that have resulted in increased sensitivity and mass accuracy have enabled significant improvements in the depth of exosomal proteome coverage. Proteomic analysis has revealed that exosomes contain a common set of membrane and cytosolic proteins, and they also contain distinct subsets of proteins that could be associated with cell type-specific functions.

Exosomes that are derived from several types of tumor cells (breast adenocarcinoma [64], colorectal cancer [72,73], mammary adenocarcinoma [74], melanoma [75,76], mesothelioma [77], and brain tumor [4,78]) have been characterized by isolation strategies including differential centrifugation, filtration, sucrose density gradient, and immunobeads, combined with mass spectrometry-based proteomic strategies such as one- and two-dimensional liquid chromatography-tandem mass spectrometry and MALDI-TOF/TOF mass spectrometry [25]. Proteomic strategies have also been used in the successful characterization of exosomes that are derived from blood [79,80,81] and various body fluids including urine [82,83], saliva [84], pleural effusions [85], and breast milk [86]. A comprehensive review of the studies that have used proteomic methods to characterize exosomes that are derived from *in vitro* sources and biological fluids has been provided by Simpson *et al*. [26].

## 7. Proteomic Analysis of the Exosome Proteome for the Development of Biomarkers

Exosomes are unique entities for biomarker analysis that have the potential to provide novel targets for therapeutic intervention. Using breast cancer cell lines that were cultured under moderate (1% O_2_) or severe (0.1% O_2_) hypoxia, King *et al*. provided evidence for the importance of understanding the hypoxic tumor phenotype that is characterized by the increased release of exosomes by hypoxic cancer cells into their microenvironment to promote their own survival and invasion [24].

Proteomic tools can be effectively used to analyze exosomes towards the elucidation of the fundamental mechanisms underlying hypoxia-mediated radiation resistance in breast cancer. In addition, the proteomic profiling of circulating tumor exosomes that can be isolated noninvasively from body fluids such as urine, plasma or serum has the potential to provide diagnostic markers for noninvasive biopsy profiling.

**Figure 4:**
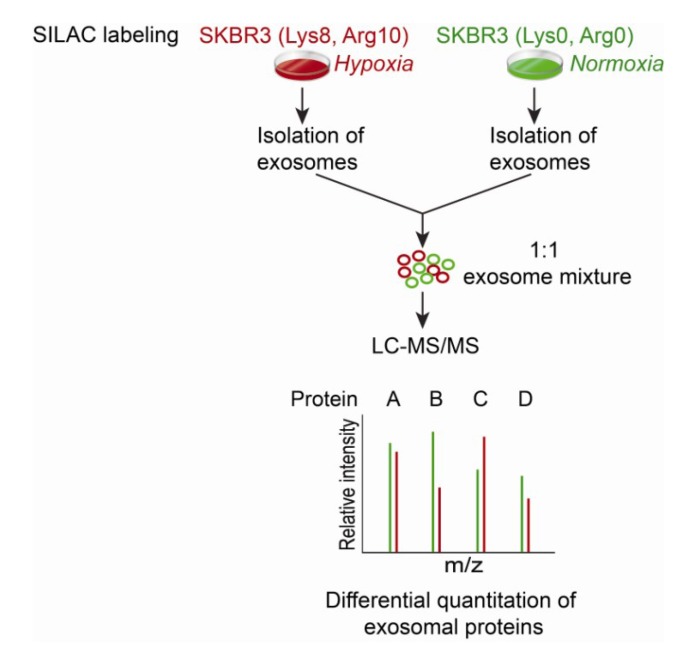
Workflow for stable isotope labeling with amino acids in cell culture (SILAC)-based quantitative proteomic profiling of exosomal proteins. Cell lines are cultured in SILAC media that has been supplemented with arginine and lysine containing ^13^C and ^15^N (Lys8, Arg10; “heavy”) or the naturally occurring ^12^C and ^14^N isotopes (Lys0, Arg0; “light”). After exposure to hypoxic or normoxic conditions, the exosomes are isolated from each cell line and are mixed at a 1:1 ratio followed by enzymatic protein digestion and liquid chromatography-tandem mass spectrometry (LC-MS/MS) analysis.

Stable isotope labeling with amino acids in cell culture (SILAC) combined with mass spectrometry is a strategy that can permit the quantitative proteomic analysis of cell culture-derived exosomes. SILAC is based on the metabolic incorporation of an isotopically “light” or “heavy” form of amino acids into proteins, the mass spectrometry analysis of which results in quantitative information regarding protein relative abundance [87]. Using this approach combined with IsoQuant [88], an in-house developed open source software package to process and quantify large proteomic datasets, we identified basic structural proteins that were directly related to exosome biogenesis, exosomal cargo recruitment and endocytosis in A549 lung cancer cells and SKBR3 breast cancer cells (unpublished observations). A schematic view of this workflow using SILAC-labeled SKBR3 cells that are cultured under hypoxic and normoxic conditions is presented in Figure 4. The data indicated that the proteomes of the exosomes directly reflected the physiological conditions and cellular contents of their parental cells, as evidenced by the significantly altered abundances of breast carcinoma-associated proteins.

## 8. Targeted Proteomic Analysis of the Exosome Proteome

The majority of the mass spectrometry-based proteomic analyses that have been described here followed a canonical “shotgun” workflow [89] whereby proteins are first digested using a specific protease, typically trypsin, and the resulting peptides are separated using reversed phase liquid chromatography. As the peptides are eluted from the reversed phase column, they are converted to gas phase ions by electrospray ionization. The analyte ions are then fragmented in the mass spectrometer, and the fragment and parent ion masses are assigned by a database searching tool to the best-matching peptide sequence in a given database. Although a wealth of information can be gleaned from these discovery phase proteomic studies, targeted proteomic assays that are most commonly based on a mass spectrometric technique called multiple (or selected) reaction monitoring (MRM) [90,91,92] using triple quadrupole mass spectrometry [93] are of increasing importance in bridging the gap between biomedical discovery and clinical implementation [94].

In typical MRM experiments, specific precursor ions representing peptides of interest are mass selected and fragmented, and the signals for only a few predefined fragment ions for each peptide of interest are monitored. This enables highly specific and quantitative multiplexed proteomic assays to be conducted. MRM-based proteomic assays have significant potential in the analysis of the exosome proteome in the context of biomarker development.

## 9. Proteomic Data Analysis

The “shotgun” or discovery-based proteomic analysis described above permits the objective identification of candidate exosomal proteins as functional biomarkers that are associated with the hypoxic microenvironments of breast tumors. Exosomal proteins, such as plasma membrane proteins, cell surface molecules, cytokines, and proteins that are localized to the cytoplasm, with significant changes in relative abundance that correlate with various disease states could be identified as significant functional biomarkers. We have a developed SILAC-based proteomics quantification software tool, termed IsoQuant, that provides users with a convenient quantification framework to calculate peptide and protein relative abundance ratios [88]. For SILAC-based proteomic studies, comparative analyses can be conducted using IsoQuant to enable the detection of exosomal proteins with differential relative abundances based on the relative intensities of the peptide ions that are derived from the “heavy” and “light”-labeled exosomal proteins in the SILAC-labeled cells. Several other mass spectrometry bioinformatic data analysis tools exist including BioWorks (Thermo Scientific), Mascot (Matrix Science), ProteinPilot (AB Sciex), and MaxQuant [95]. These and other software tools are described in detail in recent reviews [96,97,98].

**Figure 5:**
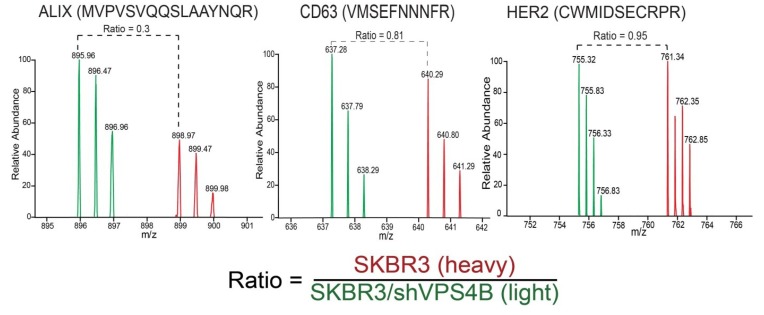
Representative SILAC MS1 spectra of Alix, CD63 and HER2 peptides with decreased relative abundance in exosomes that are derived from SKBR3 cells with ablated VPS4B expression. The relative abundance ratios were determined by IsoQuant.

Our preliminary studies have indicated that hypoxia or the decreased expression of the MVB-associated protein VPS4B in SKBR3 breast cancer cells can cause an increase in the relative abundance of exosome protein cargo in cultured cells. Figure 5 shows representative SILAC MS1 spectra of Alix (a key exosomal protein that plays an essential role in the recruitment of cargo proteins into exosomes [99,100] and ROS-mediated cell death), CD63 (a transmembrane tetraspanin family protein that is required for the fusion of exosomes with target cells and that plays a role in exosome biogenesis), and HER2 peptides that have increased relative abundance in exosomes that are derived from SKBR3 cells with ablated VPS4B expression.

Several mass spectrometry-based quantitative proteomic methods other than metabolic stable isotope labeling have been developed. Some of these methods include label-free quantification, whereby protein abundance changes are determined by measuring mass spectral peak intensities or extracted ion chromatogram peak areas and spectral counts [101], and isobaric chemical labeling using isobaric tags for relative and absolute quantification (iTRAQ) [102] or tandem mass tags (TMT) [103]. Label-free quantification methods are widely used because they are applicable to samples from any source. However, because of the requirement for the samples to be prepared and analyzed separately, the accuracy, precision, and reproducibility of label-free methods render their quantification performance inferior compared to metabolic and isobaric chemical labeling strategies [104]. Each quantitative proteomic method has its advantages and disadvantages regarding depth of proteome coverage, dynamic range, multiplexing ability, sample type compatibility, quantification accuracy, precision, and reproducibility. These factors should be taken into consideration when developing the experimental design for quantitative proteomic analyses.

**Figure 6:**
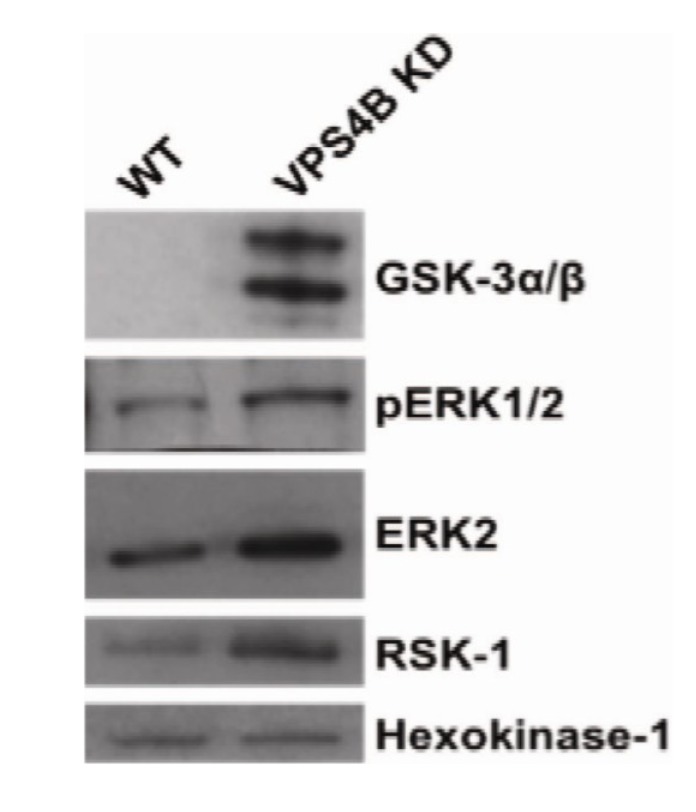
Immunoblot analysis shows accumulation of GSK3α/β and phosphorylated ERK2 signaling pathway proteins such as RSK1 in exosomes derived from SKBR3 cells with decreased VPS4B expression. Hexokinase-1 was included as an immunoblotting control for the specificity of the effect of decreased VPS4B expression. The level of hexokinase-1 expression was unchanged between exosomes from SKBR3 cells expressing WT VPS4B and cells with shRNA-mediated knockdown of VPS4B expression (VPS4B KD).

Statistical and bioinformatic analyses can be used in exosomal proteomic profiling to predict the protein interaction networks and pathways that are involved in cellular phenotype changes in response to various physiological conditions, including increased hypoxia in the breast tumor microenvironment. Results from our preliminary studies indicated the activation of ERK2 and glycogen synthase kinase-3 α and ß (GSK3α/ß) in exosomes that were isolated from SKBR3 cells with decreased levels of VPS4B expression (Figure 6). These results are consistent with data indicating that VPS4B depletion prolongs EGFR expression and signaling in EGF-treated SKBR3 cells [38].

**Figure 7:**
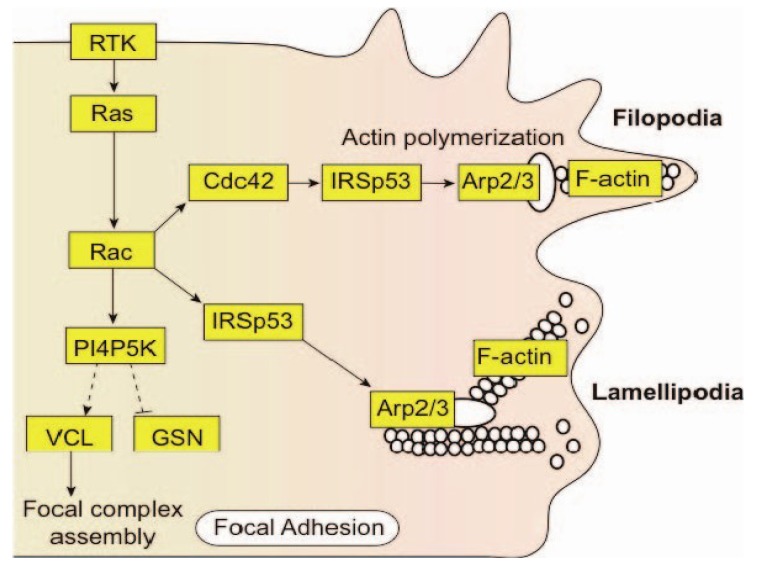
Proposed breast cancer signaling pathway based on the KEGG network pathway analysis of hypoxia-activated tumor cell migration. In EGF-stimulated SKBR3 cells with decreased VPS4B expression, the assembly of F-actin and the formation of filopodia were promoted, suggesting that the dysfunction of the MVB pathway stimulates the migration of tumor cells under hypoxic conditions.

We also conducted a Kyoto Encyclopedia of Genes and Genomes (KEGG) protein interaction network analysis of the exosomal proteome of SKBR3 cells that were cultured under hypoxic conditions, and we determined that the ErbB2/HER2 signaling pathway is highly activated. Furthermore, we found that EGF preferentially promoted the assembly of filamentous actin (F-actin) and the formation of filopodia in SKBR3 cells with decreased VPS4B expression. The EGFR signaling pathway plays a critical role in the regulation of cell proliferation, survival and differentiation in breast cancer [105,106,107]. EGFR expression is up-regulated in many human tumor tissues and the activation of EGFR signaling has been associated with aggressive types of cancer and poor responses to therapeutic treatment [108,109,110]. It has been shown that the cross-talk between EGFR and HIF-1α signaling pathways increases the resistance of breast cancer cells to apoptosis [111]. Under normoxic conditions, EGFR signaling activates the phosphoinositide 3-kinase/AKT pathway, which then increases HIF-1α levels. Taken together, our results suggest that MVB dysfunction stimulates the migration of breast tumor cells under hypoxic conditions. Based on our preliminary studies, we hypothesize that radiation-induced oxidative stress contributes to MVB dysfunction and the subsequent activation of exosome-induced tumorigenesis. Figure 7 is a proposed breast cancer signaling pathway that is based on our KEGG network analysis of hypoxia-activated tumor cell migration. This model is supported by data indicating a role for VPS4 in the localization of cell migration-related proteins at focal adhesions [112,113].

The proteomic profiling of exosomes that are released from breast cancer cells that are cultured under hypoxic conditions can yield valuable insight into the signaling pathways that are activated under these conditions. Signaling pathway and network analysis permit the visualization of large-scale proteomic datasets to enable the prioritization of functional protein targets to pursue for targeted analyses.

## 10. Conclusions

In the past few years, mass spectrometry-based proteomics methods have contributed significantly to the elucidation of the molecular composition of exosomes. It has been shown that the proteome profiles of tumor-derived exosomes are associated with the oxygenation status of patient tumors and that exosome signaling pathways are potentially targetable drivers of hypoxia-dependent intercellular signaling during tumorigenesis. Given that hypoxia is known to contribute to the radiation resistance of cancer cells and approximately half of locally advanced breast cancers have hypoxic regions where chemotherapy and radiation are less effective, the use of proteomic applications to characterize hypoxia-induced radiation resistance in breast cancer could have significant clinical utility.

This article has provided an overview of how proteomic tools can be effectively used to characterize the role of exosomes in hypoxia-mediated radiation resistance in breast cancer. The identification of an exosomal proteomic “signature” could potentially be used to enhance the efficacy of radiation cancer treatment strategies that are rendered ineffective due to hypoxic tumor microenvironments. Further advances in mass spectrometry-based proteomic technologies and the continued development of selective and efficient exosome isolation and enrichment strategies will be of great importance when elucidating the fundamental mechanisms that underlie radiation resistance in breast cancer.

## References

[1] Slamon D., Eiermann W., Robert N., Pienkowski T., Martin M., Press M., Mackey J., Glaspy J., Chan A., Pawlicki M. (2011). Adjuvant trastuzumab in HER2-positive breast cancer. N. Engl. J. Med..

[2] Vaupel P., Briest S., Hockel M. (2002). Hypoxia in breast cancer: Pathogenesis, characterization and biological/therapeutic implications. Wien. Med. Wochenschr..

[3] Vaupel P., Mayer A. (2007). Hypoxia in cancer: Significance and impact on clinical outcome. Cancer Metastasis Rev..

[4] Kucharzewska P., Christianson H.C., Welch J.E., Svensson K.J., Fredlund E., Ringner M., Morgelin M., Bourseau-Guilmain E., Bengzon J., Belting M. (2013). Exosomes reflect the hypoxic status of glioma cells and mediate hypoxia-dependent activation of vascular cells during tumor development. Proc. Natl. Acad. Sci.USA.

[5] Garnier D., Jabado N., Rak J. (2012). Extracellular vesicles as prospective carriers of oncogenic protein signatures in adult and paediatric brain tumours. Proteomics.

[6] Bellingham S.A., Guo B.B., Coleman B.M., Hill A.F. (2012). Exosomes: Vehicles for the transfer of toxic proteins associated with neurodegenerative diseases?. Front. Physiol..

[7] Yang M., Chen J., Su F., Yu B., Lin L., Liu Y., Huang J.D., Song E. (2011). Microvesicles secreted by macrophages shuttle invasion-potentiating microRNAs into breast cancer cells. Mol. Cancer.

[8] Park J.E., Tan H.S., Datta A., Lai R.C., Zhang H., Meng W., Lim S.K., Sze S.K. (2011). Hypoxic tumor cell modulates its microenvironment to enhance angiogenic and metastatic potential by secretion of proteins and exosomes. Mol. Cell. Proteomics.

[9] Subra C., Grand D., Laulagnier K., Stella A., Lambeau G., Paillasse M., de Medina P., Monsarrat B., Perret B., Silvente-Poirot S. (2010). Exosomes account for vesicle-mediated transcellular transport of activatable phospholipases and prostaglandins. J. Lipid Res..

[10] Taylor D.D., Gercel-Taylor C. (2005). Tumour-derived exosomes and their role in cancer-associated T-cell signalling defects. Br. J. Cancer.

[11] Azmi A.S., Bao B., Sarkar F.H. (2013). Exosomes in cancer development, metastasis, and drug resistance: A comprehensive review. Cancer Metastasis Rev..

[12] EL Andaloussi S., Mager I., Breakefield X.O., Wood M.J. (2013). Extracellular vesicles: Biology and emerging therapeutic opportunities. Nat. Rev. Drug Discov..

[13] Simona F., Laura S., Simona T., Riccardo A. (2013). Contribution of proteomics to understanding the role of tumor-derived exosomes in cancer progression: State of the art and new perspectives. Proteomics.

[14] Martins V.R., Dias M.S., Hainaut P. (2013). Tumor-cell-derived microvesicles as carriers of molecular information in cancer. Curr. Opin. Oncol..

[15] Yu X., Harris S.L., Levine A.J. (2006). The regulation of exosome secretion: A novel function of the p53 protein. Cancer Res..

[16] Lehmann B.D., Paine M.S., Brooks A.M., McCubrey J.A., Renegar R.H., Wang R., Terrian D.M. (2008). Senescence-associated exosome release from human prostate cancer cells. Cancer Res..

[17] Lespagnol A., Duflaut D., Beekman C., Blanc L., Fiucci G., Marine J.C., Vidal M., Amson R., Telerman A. (2008). Exosome secretion, including the DNA damage-induced p53-dependent secretory pathway, is severely compromised in TSAP6/Steap3-null mice. Cell Death Differ..

[18] Trajkovic K., Hsu C., Chiantia S., Rajendran L., Wenzel D., Wieland F., Schwille P., Brugger B., Simons M. (2008). Ceramide triggers budding of exosome vesicles into multivesicular endosomes. Science.

[19] Savina A., Fader C.M., Damiani M.T., Colombo M.I. (2005). Rab11 promotes docking and fusion of multivesicular bodies in a calcium-dependent manner. Traffic.

[20] Parolini I., Federici C., Raggi C., Lugini L., Palleschi S., de Milito A., Coscia C., Iessi E., Logozzi M., Molinari A. (2009). Microenvironmental pH is a key factor for exosome traffic in tumor cells. J. Biol. Chem..

[21] Svensson K.J., Kucharzewska P., Christianson H.C., Skold S., Lofstedt T., Johansson M.C., Morgelin M., Bengzon J., Ruf W., Belting M. (2011). Hypoxia triggers a proangiogenic pathway involving cancer cell microvesicles and PAR-2-mediated heparin-binding EGF signaling in endothelial cells. Proc. Natl. Acad. Sci. USA.

[22] Orriss I.R., Knight G.E., Utting J.C., Taylor S.E., Burnstock G., Arnett T.R. (2009). Hypoxia stimulates vesicular ATP release from rat osteoblasts. J. Cell. Physiol..

[23] Wysoczynski M., Ratajczak M.Z. (2009). Lung cancer secreted microvesicles: Underappreciated modulators of microenvironment in expanding tumors. Int. J. Cancer.

[24] King H.W., Michael M.Z., Gleadle J.M. (2012). Hypoxic enhancement of exosome release by breast cancer cells. BMC Cancer.

[25] Simpson R.J., Jensen S.S., Lim J.W. (2008). Proteomic profiling of exosomes: Current perspectives. Proteomics.

[26] Simpson R.J., Lim J.W., Moritz R.L., Mathivanan S. (2009). Exosomes: Proteomic insights and diagnostic potential. Expert Rev. Proteomics.

[27] Ward C., Langdon S.P., Mullen P., Harris A.L., Harrison D.J., Supuran C.T., Kunkler I.H. (2013). New strategies for targeting the hypoxic tumour microenvironment in breast cancer. Cancer Treat. Rev..

[28] Gatenby R.A., Smallbone K., Maini P.K., Rose F., Averill J., Nagle R.B., Worrall L., Gillies R.J. (2007). Cellular adaptations to hypoxia and acidosis during somatic evolution of breast cancer. Br. J. Cancer.

[29] Bristow R.G., Hill R.P. (2008). Hypoxia and metabolism: Hypoxia, DNA repair and genetic instability. Nat. Rev. Cancer.

[30] Thomlinson R.H., Gray L.H. (1955). The histological structure of some human lung cancers and the possible implications for radiotherapy. Br. J. Cancer.

[31] Bindra R.S., Crosby M.E., Glazer P.M. (2007). Regulation of DNA repair in hypoxic cancer cells. Cancer Metastasis Rev..

[32] Yoshimura M., Itasaka S., Harada H., Hiraoka M. (2013). Microenvironment and radiation therapy. Biomed. Res. Int..

[33] Harada H. (2011). How can we overcome tumor hypoxia in radiation therapy?. J. Radiat. Res..

[34] Brown J.M. (2000). Exploiting the hypoxic cancer cell: Mechanisms and therapeutic strategies. Mol. Med. Today.

[35] Brown J.M., Wilson W.R. (2004). Exploiting tumour hypoxia in cancer treatment. Nat. Rev. Cancer.

[36] Ren Y., Hao P., Dutta B., Cheow E.S., Sim K.H., Gan C.S., Lim S.K., Sze S.K. (2013). Hypoxia modulates A431 cellular pathways association to tumor radioresistance and enhanced migration revealed by comprehensive proteomic and functional studies. Mol. Cell. Proteomics.

[37] Sirbu B.M., Cortez D. (2013). DNA damage response: Three levels of DNA repair regulation. Cold Spring Harb. Perspect. Biol..

[38] Lin H.H., Li X., Chen J.L., Sun X., Cooper F.N., Chen Y.R., Zhang W., Chung Y., Li A., Cheng C.T. (2012). Identification of an AAA ATPase VPS4B-dependent pathway that modulates epidermal growth factor receptor abundance and signaling during hypoxia. Mol. Cell. Biol..

[39] Liu J., Zhang J., Wang X., Li Y., Chen Y., Li K., Zhang J., Yao L., Guo G. (2010). HIF-1 and NDRG2 contribute to hypoxia-induced radioresistance of cervical cancer Hela cells. Exp. Cell. Res..

[40] Moeller B.J., Dewhirst M.W. (2006). HIF-1 and tumour radiosensitivity. Br. J. Cancer.

[41] Kaidar-Person O., Lai C., Kuten A., Belkacemi Y. (2013). “The Infinite Maze” of breast cancer, signaling pathways and radioresistance. Breast.

[42] Grosso S., Doyen J., Parks S.K., Bertero T., Paye A., Cardinaud B., Gounon P., Lacas-Gervais S., Noel A., Pouyssegur J. (2013). MiR-210 promotes a hypoxic phenotype and increases radioresistance in human lung cancer cell lines. Cell Death Dis..

[43] Potiron V.A., Abderrhamani R., Giang E., Chiavassa S., di Tomaso E., Maira S.M., Paris F., Supiot S. (2013). Radiosensitization of prostate cancer cells by the dual PI3K/mTOR inhibitor BEZ235 under normoxic and hypoxic conditions. Radiother. Oncol..

[44] Tamara Marie-Egyptienne D., Lohse I., Hill R.P. (2012). Cancer stem cells, the epithelial to mesenchymal transition (EMT) and radioresistance: Potential role of hypoxia. Cancer Lett..

[45] Eldh M., Ekstrom K., Valadi H., Sjostrand M., Olsson B., Jernas M., Lotvall J. (2010). Exosomes communicate protective messages during oxidative stress; possible role of exosomal shuttle RNA. PLoS One.

[46] Vlassov A.V., Magdaleno S., Setterquist R., Conrad R. (2012). Exosomes: Current knowledge of their composition, biological functions, and diagnostic and therapeutic potentials. Biochim. Biophys. Acta.

[47] Thery C., Zitvogel L., Amigorena S. (2002). Exosomes: Composition, biogenesis and function. Nat. Rev. Immunol..

[48] Conde-Vancells J., Rodriguez-Suarez E., Embade N., Gil D., Matthiesen R., Valle M., Elortza F., Lu S.C., Mato J.M., Falcon-Perez J.M. (2008). Characterization and comprehensive proteome profiling of exosomes secreted by hepatocytes. J. Proteome Res..

[49] Wubbolts R., Leckie R.S., Veenhuizen P.T., Schwarzmann G., Mobius W., Hoernschemeyer J., Slot J.W., Geuze H.J., Stoorvogel W. (2003). Proteomic and biochemical analyses of human B cell-derived exosomes. J. Biol. Chem..

[50] Balaj L., Lessard R., Dai L., Cho Y.J., Pomeroy S.L., Breakefield X.O., Skog J. (2011). Tumour microvesicles contain retrotransposon elements and amplified oncogene sequences. Nat. Commun..

[51] Gibbings D.J., Ciaudo C., Erhardt M., Voinnet O. (2009). Multivesicular bodies associate with components of miRNA effector complexes and modulate miRNA activity. Nat. Cell Biol..

[52] Lee Y.S., Shibata Y., Malhotra A., Dutta A. (2009). A novel class of small RNAs: tRNA-derived RNA fragments (tRFs). Genes Dev..

[53] Haussecker D., Huang Y., Lau A., Parameswaran P., Fire A.Z., Kay M.A. (2010). Human tRNA-derived small RNAs in the global regulation of RNA silencing. RNA.

[54] Thomas S.N., Wan Y., Liao Z., Hanson P.I., Yang A.J. (2011). Stable isotope labeling with amino acids in cell culture based mass spectrometry approach to detect transient protein interactions using substrate trapping. Anal. Chem..

[55] Liao Z., Thomas S.N., Wan Y., Lin H.H., Ann D.K., Yang A.J. (2013). An internal standard-assisted synthesis and degradation proteomic approach reveals the potential linkage between VPS4B depletion and activation of fatty acid beta-oxidation in breast cancer cells. Int. J. Proteomics.

[56] Battke C., Ruiss R., Welsch U., Wimberger P., Lang S., Jochum S., Zeidler R. (2011). Tumour exosomes inhibit binding of tumour-reactive antibodies to tumour cells and reduce ADCC. Cancer Immunol. Immunother..

[57] Ciravolo V., Huber V., Ghedini G.C., Venturelli E., Bianchi F., Campiglio M., Morelli D., Villa A., Della Mina P., Menard S. (2012). Potential role of HER2-overexpressing exosomes in countering trastuzumab-based therapy. J. Cell. Physiol..

[58] Thery C., Amigorena S., Raposo G., Clayton A. (2006). Isolation and characterization of exosomes from cell culture supernatants and biological fluids. Curr. Protoc. Cell Biol..

[59] Thery C., Boussac M., Veron P., Ricciardi-Castagnoli P., Raposo G., Garin J., Amigorena S. (2001). Proteomic analysis of dendritic cell-derived exosomes: A secreted subcellular compartment distinct from apoptotic vesicles. J. Immunol..

[60] Thery C., Regnault A., Garin J., Wolfers J., Zitvogel L., Ricciardi-Castagnoli P., Raposo G., Amigorena S. (1999). Molecular characterization of dendritic cell-derived exosomes. J. Cell Biol..

[61] Cheruvanky A., Zhou H., Pisitkun T., Kopp J.B., Knepper M.A., Yuen P.S., Star R.A. (2007). Rapid isolation of urinary exosomal biomarkers using a nanomembrane ultrafiltration concentrator. Am. J. Physiol. Renal Physiol..

[62] de Bock K., Mazzone M., Carmeliet P. (2011). Antiangiogenic therapy, hypoxia, and metastasis: Risky liaisons, or not?. Nat. Rev. Clin. Oncol..

[63] Marleau A.M., Chen C.S., Joyce J.A., Tullis R.H. (2012). Exosome removal as a therapeutic adjuvant in cancer. J. Transl. Med..

[64] Koga K., Matsumoto K., Akiyoshi T., Kubo M., Yamanaka N., Tasaki A., Nakashima H., Nakamura M., Kuroki S., Tanaka M. (2005). Purification, characterization and biological significance of tumor-derived exosomes. Anticancer Res..

[65] Nahta R., Yu D., Hung M.C., Hortobagyi G.N., Esteva F.J. (2006). Mechanisms of disease: Understanding resistance to HER2-targeted therapy in human breast cancer. Nat. Clin. Pract. Oncol..

[66] Abdel-Razeq H., Marei L. (2011). Current neoadjuvant treatment options for HER2-positive breast cancer. Biologics.

[67] von Minckwitz G., Loibl S., Untch M. (2012). What is the current standard of care for anti-HER2 neoadjuvant therapy in breast cancer?. Oncology.

[68] Aung T., Chapuy B., Vogel D., Wenzel D., Oppermann M., Lahmann M., Weinhage T., Menck K., Hupfeld T., Koch R. (2011). Exosomal evasion of humoral immunotherapy in aggressive B-cell lymphoma modulated by ATP-binding cassette transporter A3. Proc. Natl. Acad. Sci. USA.

[69] Safaei R., Larson B.J., Cheng T.C., Gibson M.A., Otani S., Naerdemann W., Howell S.B. (2005). Abnormal lysosomal trafficking and enhanced exosomal export of cisplatin in drug-resistant human ovarian carcinoma cells. Mol. Cancer Ther..

[70] Chen K.G., Valencia J.C., Lai B., Zhang G., Paterson J.K., Rouzaud F., Berens W., Wincovitch S.M., Garfield S.H., Leapman R.D. (2006). Melanosomal sequestration of cytotoxic drugs contributes to the intractability of malignant melanomas. Proc. Natl. Acad. Sci. USA.

[71] Shedden K., Xie X.T., Chandaroy P., Chang Y.T., Rosania G.R. (2003). Expulsion of small molecules in vesicles shed by cancer cells: Association with gene expression and chemosensitivity profiles. Cancer Res..

[72] Choi D.S., Lee J.M., Park G.W., Lim H.W., Bang J.Y., Kim Y.K., Kwon K.H., Kwon H.J., Kim K.P., Gho Y.S. (2007). Proteomic analysis of microvesicles derived from human colorectal cancer cells. J. Proteome Res..

[73] Ji H., Greening D.W., Barnes T.W., Lim J.W., Tauro B.J., Rai A., Xu R., Adda C., Mathivanan S., Zhao W. (2013). Proteome profiling of exosomes derived from human primary and metastatic colorectal cells reveal differential expression of key metastatic factors and signal transduction components. Proteomics.

[74] Wolfers J., Lozier A., Raposo G., Regnault A., Thery C., Masurier C., Flament C., Pouzieux S., Faure F., Tursz T. (2001). Tumor-derived exosomes are a source of shared tumor rejection antigens for CTL cross-priming. Nat. Med..

[75] Craven R.A., Totty N., Harnden P., Selby P.J., Banks R.E. (2002). Laser capture microdissection and two-dimensional polyacrylamide gel electrophoresis: Evaluation of tissue preparation and sample limitations. Am. J. Pathol..

[76] Marton A., Vizler C., Kusz E., Temesfoi V., Szathmary Z., Nagy K., Szegletes Z., Varo G., Siklos L., Katona R.L. (2012). Melanoma cell-derived exosomes alter macrophage and dendritic cell functions *in vitro*. Immunol. Lett..

[77] Hegmans J.P., Bard M.P., Hemmes A., Luider T.M., Kleijmeer M.J., Prins J.B., Zitvogel L., Burgers S.A., Hoogsteden H.C., Lambrecht B.N. (2004). Proteomic analysis of exosomes secreted by human mesothelioma cells. Am. J. Pathol..

[78] Graner M.W., Alzate O., Dechkovskaia A.M., Keene J.D., Sampson J.H., Mitchell D.A., Bigner D.D. (2009). Proteomic and immunologic analyses of brain tumor exosomes. FASEB J..

[79] Caby M.P., Lankar D., Vincendeau-Scherrer C., Raposo G., Bonnerot C. (2005). Exosomal-like vesicles are present in human blood plasma. Int. Immunol..

[80] Looze C., Yui D., Leung L., Ingham M., Kaler M., Yao X., Wu W.W., Shen R.F., Daniels M.P., Levine S.J. (2009). Proteomic profiling of human plasma exosomes identifies PPARgamma as an exosome-associated protein. Biochem. Biophys. Res. Commun..

[81] Sabapatha A., Gercel-Taylor C., Taylor D.D. (2006). Specific isolation of placenta-derived exosomes from the circulation of pregnant women and their immunoregulatory consequences. Am. J. Reprod. Immunol..

[82] Gonzales P.A., Pisitkun T., Hoffert J.D., Tchapyjnikov D., Star R.A., Kleta R., Wang N.S., Knepper M.A. (2009). Large-scale proteomics and phosphoproteomics of urinary exosomes. J. Am. Soc. Nephrol..

[83] Nilsson J., Skog J., Nordstrand A., Baranov V., Mincheva-Nilsson L., Breakefield X.O., Widmark A. (2009). Prostate cancer-derived urine exosomes: A novel approach to biomarkers for prostate cancer. Br. J. Cancer.

[84] Gonzalez-Begne M., Lu B., Han X., Hagen F.K., Hand A.R., Melvin J.E., Yates J.R. (2009). Proteomic analysis of human parotid gland exosomes by multidimensional protein identification technology (MudPIT). J. Proteome Res..

[85] Andre F., Schartz N.E., Movassagh M., Flament C., Pautier P., Morice P., Pomel C., Lhomme C., Escudier B., Le Chevalier T. (2002). Malignant effusions and immunogenic tumour-derived exosomes. Lancet.

[86] Admyre C., Johansson S.M., Qazi K.R., Filen J.J., Lahesmaa R., Norman M., Neve E.P., Scheynius A., Gabrielsson S. (2007). Exosomes with immune modulatory features are present in human breast milk. J. Immunol..

[87] Ong S.E., Blagoev B., Kratchmarova I., Kristensen D.B., Steen H., Pandey A., Mann M. (2002). Stable isotope labeling by amino acids in cell culture, SILAC, as a simple and accurate approach to expression proteomics. Mol. Cell. Proteomics.

[88] Liao Z., Wan Y., Thomas S.N., Yang A.J. (2012). IsoQuant: A software tool for stable isotope labeling by amino acids in cell culture-based mass spectrometry quantitation. Anal. Chem..

[89] Aebersold R., Mann M. (2003). Mass spectrometry-based proteomics. Nature.

[90] Picotti P., Aebersold R. (2012). Selected reaction monitoring-based proteomics: Workflows, potential, pitfalls and future directions. Nat. Methods.

[91] Anderson L., Hunter C.L. (2006). Quantitative mass spectrometric multiple reaction monitoring assays for major plasma proteins. Mol. Cell. Proteomics.

[92] Lange V., Picotti P., Domon B., Aebersold R. (2008). Selected reaction monitoring for quantitative proteomics: A tutorial. Mol. Syst. Biol..

[93] Yost R.A., Enke C.G. (1979). Triple quadrupole mass spectrometry for direct mixture analysis and structure elucidation. Anal. Chem..

[94] Gillette M.A., Carr S.A. (2013). Quantitative analysis of peptides and proteins in biomedicine by targeted mass spectrometry. Nat. Methods.

[95] Cox J., Mann M. (2008). MaxQuant enables high peptide identification rates, individualized p.p.b.-range mass accuracies and proteome-wide protein quantification. Nat. Biotechnol..

[96] Vaudel M., Sickmann A., Martens L. (2012). Current methods for global proteome identification. Expert Rev. Proteomics.

[97] Eng J.K., Searle B.C., Clauser K.R., Tabb D.L. (2011). A face in the crowd: Recognizing peptides through database search. Mol. Cell. Proteomics.

[98] Matthiesen R., Carvalho A.S. (2010). Methods and algorithms for relative quantitative proteomics by mass spectrometry. Methods Mol. Biol..

[99] Baietti M.F., Zhang Z., Mortier E., Melchior A., Degeest G., Geeraerts A., Ivarsson Y., Depoortere F., Coomans C., Vermeiren E. (2012). Syndecan-syntenin-ALIX regulates the biogenesis of exosomes. Nat. Cell Biol..

[100] Hurley J.H., Odorizzi G. (2012). Get on the exosome bus with ALIX. Nat. Cell Biol..

[101] Old W.M., Meyer-Arendt K., Aveline-Wolf L., Pierce K.G., Mendoza A., Sevinsky J.R., Resing K.A., Ahn N.G. (2005). Comparison of label-free methods for quantifying human proteins by shotgun proteomics. Mol. Cell. Proteomics.

[102] Ross P.L., Huang Y.N., Marchese J.N., Williamson B., Parker K., Hattan S., Khainovski N., Pillai S., Dey S., Daniels S. (2004). Multiplexed protein quantitation in *Saccharomyces cerevisiae* using amine-reactive isobaric tagging reagents. Mol. Cell. Proteomics.

[103] Thompson A., Schafer J., Kuhn K., Kienle S., Schwarz J., Schmidt G., Neumann T., Johnstone R., Mohammed A.K., Hamon C. (2003). Tandem mass tags: A novel quantification strategy for comparative analysis of complex protein mixtures by MS/MS. Anal. Chem..

[104] Li Z., Adams R.M., Chourey K., Hurst G.B., Hettich R.L., Pan C. (2012). Systematic comparison of label-free, metabolic labeling, and isobaric chemical labeling for quantitative proteomics on LTQ Orbitrap Velos. J. Proteome Res..

[105] Chrysogelos S.A., Dickson R.B. (1994). EGF receptor expression, regulation, and function in breast cancer. Breast Cancer Res. Treat..

[106] Chrysogelos S.A., Yarden R.I., Lauber A.H., Murphy J.M. (1994). Mechanisms of EGF receptor regulation in breast cancer cells. Breast Cancer Res. Treat..

[107] Danielsen A.J., Maihle N.J. (2002). The EGF/ErbB receptor family and apoptosis. Growth Factors.

[108] Bucci B., D'Agnano I., Botti C., Mottolese M., Carico E., Zupi G., Vecchione A. (1997). EGF-R expression in ductal breast cancer: Proliferation and prognostic implications. Anticancer Res..

[109] Buchholz T.A., Tu X., Ang K.K., Esteva F.J., Kuerer H.M., Pusztai L., Cristofanilli M., Singletary S.E., Hortobagyi G.N., Sahin A.A. (2005). Epidermal growth factor receptor expression correlates with poor survival in patients who have breast carcinoma treated with doxorubicin-based neoadjuvant chemotherapy. Cancer.

[110] Navolanic P.M., Steelman L.S., McCubrey J.A. (2003). EGFR family signaling and its association with breast cancer development and resistance to chemotherapy. Int. J. Oncol..

[111] Peng X.H., Karna P., Cao Z., Jiang B.H., Zhou M., Yang L. (2006). Cross-talk between epidermal growth factor receptor and hypoxia-inducible factor-1alpha signal pathways increases resistance to apoptosis by up-regulating survivin gene expression. J. Biol. Chem..

[112] Tu C., Ortega-Cava C.F., Winograd P., Stanton M.J., Reddi A.L., Dodge I., Arya R., Dimri M., Clubb R.J., Naramura M. (2010). Endosomal-sorting complexes required for transport (ESCRT) pathway-dependent endosomal traffic regulates the localization of active Src at focal adhesions. Proc. Natl. Acad. Sci. USA.

[113] Tu C., Ahmad G., Mohapatra B., Bhattacharyya S., Ortega-Cava C.F., Chung B.M., Wagner K.U., Raja S.M., Naramura M., Band V. (2011). ESCRT proteins: Double-edged regulators of cellular signaling. Bioarchitecture.

